# Dataset of Vietnamese preschool teachers' readiness towards implementing STEAM activities and projects

**DOI:** 10.1016/j.dib.2022.108821

**Published:** 2022-12-12

**Authors:** Thi-Lam Bui, Thi-Tham Tran, Thanh-Huong Nguyen, Luyen Nguyen-Thi, Viet-Nhi Tran, Ut Phuong Dang, Manh-Tuan Nguyen, Anh-Duc Hoang

**Affiliations:** a Faculty of Early Childhood Education, Hanoi National University of Education, Hanoi 100000, Vietnam; b Faculty of Preschool Education, University of Education, Hue University 530000, Vietnam; c Faculty of Pedagogy, Hanoi Metropolitan University, Hanoi 100000, Vietnam; d EdLab Asia Educational Research and Development Centre, Hanoi 100000, Vietnam

**Keywords:** Early childhood education, Teacher education, STEAM education, Culture education, Place-based education, Vietnam

## Abstract

STEAM education has significant impacts on improving preschoolers' learning engagement, cognition, confidence, and innovative thinking skills. The integration of content about local culture into STEAM projects also triggers students' awareness and engagement with surrounding communities and societies. However, teachers' competencies and willingness to implement STEAM activities are irreplaceable in determining STEM projects' frequency and effectiveness. This dataset surveyed 754 preschool teachers across ten cities and provinces in Vietnam from 8 Apr 2022 to 15 May 2022. The dataset includes seven main sections: (i) Demographics; (ii) Teachers' know-how about STEAM education; (iii) Teachers' competencies; (iv) Conditions to implement STEAM projects; (v) School policies; (vi) Local culture factors integrated with STEAM projects; and (vii) Teachers' willingness to implement STEAM education.

**Specifications Table**

**Table:** 

Subject	Education
Specific subject area	Early Childhood Education, Teacher Development
Type of data	Table
How data were acquired	Data was gathered using an online survey (Google Form).
Data format	Raw Analyzed
Description of data collection	An online survey has been distributed throughout randomly selected preschools (from 23 Apr 2022 to 15 May 2022) in 10 provinces and cities across Vietnam. The researchers emailed the survey to randomly selected principals and asked them to forward it to their teachers on a voluntary basis. Within the consent form at the beginning of the survey, teachers also get informed that their participation is voluntary.
Data source location	Ten cities and provinces across Vietnam: Hanoi (Latitude 21°1′N, Longitude 105°5′E), Lai Chau (Latitude 22°23′N, Longitude 103°27′E), Phu Tho (Latitude 21°16′N, Longitude 105°12′E), Hue (Latitude 16°27′N, Longitude 107°35′E), Da Nang (Latitude 16°2′N, Longitude 108°12′E), DakLak (Latitude 12°42′N, Longitude 108°14′E), Ho Chi Minh city (Latitude 10°45′N, Longitude 106°39′E), Dong Nai (Latitude 10°57′N, Longitude 106°51′E), Dong Thap (Latitude 10°40′N, Longitude 105°30′E), Can Tho (Latitude 10°2′N, Longitude 105°44′E).
Data accessibility	Repository name: Harvard Dataverse Data identification number: VOSJWK Direct URL to data: 10.7910/DVN/VOSJWK

## Value of the Data

• This dataset captures Vietnamese preschool teachers' perceptions, capabilities, and their willingness to implement STEAM activities and projects.
• This dataset captures Vietnamese preschools' policies and infrastructure that enable or inhibit teachers to implement STEAM activities and projects.
• This dataset provides a rounded picture of Vietnamese preschool teachers’ readiness to integrate local cultural values and content into STEAM activities and projects.
• This dataset would be helpful for future reference among educational policymakers and practitioners to perform comparative studies about the readiness and gaps in implementing STEAM education activities in the ECE context.

## 1 Data Description

This dataset focuses on Vietnamese preschool teachers' perceived knowledge and competency to implement STEAM activities, as well as infrastructure, policy, and local educational content towards integrated STEAM activities. The dataset was conducted through an online survey using Google Form, from 8 Apr 2022 to 15 May 2022. Participants from randomly selected preschools across ten cities and provinces in Vietnam participated in this project on a voluntary basis. After receiving all the responses and clarifying the data with 754 validated observations, we exported them as an Excel file (including both *xlsx and *csv formats) for importing to SPSS 20. The excel file (*xlsx) reposited in Harvard Dataverse [1] includes two sheets: (i) the complete questionnaires with detailed parameters and (ii) the raw dataset. The questionnaires included seven demographic questions and 55 measurement items. Table 1 contains six fields about the demographics of 754 respondents, except their years of experience, which can be found in the excel file (column A, sheet 2).

**Table 1:** Demographics of respondents (*N* = 754).

Variable	Frequency	Percent
***Qualification***	***754***	***100***
Postgraduates	29	3.8
Bachelor's degree	458	60.7
College degree (3 years of training)	114	15.1
Intermediate degree (2 years of training)	153	20.3
***Region***	***754***	***100***
Urban area	458	60.7
Rural area	214	28.4
Mountainous area	82	10.9
***Type of school***	***754***	***100***
Public preschool	537	71.2
Private preschool	186	24.7
Independent child-care group	18	2.4
Family child-care group	13	1.7
***Position***	***754***	***100***
Principal/ vice-principal	116	15.4
Professional group leader/ vice leader	129	17.1
Teacher	509	67.5
***Training and working experiences related to STEAM***	***754***	***100***
I have NEITHER participated in STEAM education training courses NOR ORGANIZED STEAM activities	366	48.5
I have NOT attended the training yet, but I HAVE ORGANIZED STEAM activities	84	11.1
I have been trained but I'm NOT CONFIDENT in organizing STEAM activities	176	23.3
I have been trained but I'm CONFIDENT in organizing STEAM activities	128	17.0
***I was trained by…***	***754***	***100***
Provincial-level Department of Education and training	83	11.0
District-level Department of Education and training	128	16.9
My preschool	130	17.2
Others	44	5.8
Not trained	369	48.9

The main questionnaires with 55 items consist of six sections: (i) Teachers' know-how about STEAM education (9 items); (ii) Teachers' competencies to implement STEAM projects for Preschoolers (17 items); (iii) Conditions to implement STEAM projects (8 items); (iv) School policies and leadership toward the implementation of the STEAM project (6 items); (v) Local culture factors integrated with STEAM projects (11 items); and (vi) Teachers' willingness to implement STEAM activities/ projects for preschoolers (4 items). We used various Likert scales of four, five, and six to eliminate the common method bias.

STEAM education is an ideal approach that fits preschool children's learning styles. STEAM activities and projects stimulate children's interest in learning and positively impact cognitive development and problem-solving capacity, building confidence, dynamism, and innovative thinking for children [2,3]. Preschool teachers play an essential role in organizing STEAM education activities, especially in aligning the targets of those activities with the expected learning outcomes for the children, the school, and the region [3,4]. Regarding the case of Vietnam, STEM/STEAM education is not a new research topic for scholars who focus on elementary, lower secondary, or upper secondary education [5]. However, there was little attention on STEAM education in Early Childhood Education (ECE). Therefore, based on the prior findings of research on STEAM education in ECE [6], [7], [8], [9], [10], we came up with the questionnaire for this study. The first set of questions describes Vietnamese preschool teachers' understanding of STEAM education as well as their competencies in implementing STEAM activities or projects for preschoolers (Table 2). The participants were asked to self-evaluate their levels of understanding of various STEM concepts and approaches (from No. A001 to A009), as well as their ability to design the lesson plan, prepare the environment, implement, assess, and enhance preschoolers' learning processes (from No. B001 to B017), as follow:

• *A001 – A006:* Teachers' know-how about STEAM education *philosophy and contents*
• *A007 – A009:* Teachers' know-how about STEAM education *methods*
• *B001 – B004:* Teachers' competencies to planning STEAM projects for Preschoolers
• *B004 – B008:* Teachers' competencies to building an environment for STEAM projects for Preschoolers
• *B009 – B013:* Teachers' competencies to organizing STEAM projects for Preschoolers
• *B014 – B017:* Teachers' competencies to evaluating the implementation of STEAM projects for Preschoolers

**Table 2:** Descriptive statistic of Teachers' STEAM know-how and competencies (*N* = 754).

Variables	Range	Min	Max	Mean	Std. Error	Std. Deviation	Variance
**A -** Teachers' know-how about STEAM education							
A001	3.0	1.0	4.0	2.756	.032	.886	.785
A002	3.0	1.0	4.0	2.927	.030	.830	.689
A003	3.0	1.0	4.0	2.874	.031	.837	.700
A004	3.0	1.0	4.0	2.837	.030	.825	.681
A005	3.0	1.0	4.0	2.993	.031	.863	.745
A006	3.0	1.0	4.0	3.084	.031	.842	.709
A007	3.0	1.0	4.0	2.731	.031	.847	.718
A008	3.0	1.0	4.0	2.724	.031	.858	.737
A009	3.0	1.0	4.0	2.723	.032	.872	.761
**B -** Teachers' competencies to implement STEAM projects for Preschoolers							
B001	5.0	1.0	6.0	2.995	.044	1.193	1.424
B002	5.0	1.0	6.0	3.019	.045	1.232	1.519
B003	5.0	1.0	6.0	3.008	.044	1.217	1.482
B004	5.0	1.0	6.0	3.127	.044	1.212	1.468
B005	5.0	1.0	6.0	3.065	.044	1.202	1.445
B006	5.0	1.0	6.0	3.207	.045	1.236	1.527
B007	5.0	1.0	6.0	3.293	.046	1.257	1.581
B008	5.0	1.0	6.0	3.281	.046	1.253	1.570
B009	5.0	1.0	6.0	3.340	.0460	1.262	1.592
B010	5.0	1.0	6.0	3.361	.046	1.257	1.580
B011	5.0	1.0	6.0	3.235	.045	1.223	1.497
B012	5.0	1.0	6.0	3.184	.045	1.227	1.505
B013	5.0	1.0	6.0	3.241	.045	1.229	1.511
B014	5.0	1.0	6.0	3.322	.045	1.239	1.536
B015	5.0	1.0	6.0	3.365	.045	1.243	1.547
B016	5.0	1.0	6.0	3.280	.045	1.224	1.498
B017	5.0	1.0	6.0	3.263	.046	1.223	1.495

Regarding the case of the Vietnamese early childhood education system, teachers have significant autonomy to diversify the children's learning experience through integrated pedagogical approaches and enriched content [6]. Table 3 reports Vietnamese preschools' readiness (from No. C001 to C008) and organizational supportiveness (from No. D001 to D006) to implement STEAM activities and projects. Those elements are essential to foster teachers' willingness to participate in the STEAM designing and planning process and the effectiveness of those activities [7].

**Table 3:** Descriptive statistic of infrastructure, policy, and management-related factors toward implementing STEAM projects (*N* = 754).

Variables	Range	Min	Max	Mean	Std. Error	Std. Deviation	Variance
**C -** Conditions to implement STEAM projects							
C001	4.0	1.0	5.0	2.757	.039	1.081	1.169
C002	4.0	1.0	5.0	2.755	.039	1.068	1.142
C003	4.0	1.0	5.0	2.890	.040	1.085	1.176
C004	4.0	1.0	5.0	2.910	.039	1.076	1.158
C005	4.0	1.0	5.0	2.720	.038	1.054	1.110
C006	4.0	1.0	5.0	2.779	.039	1.072	1.150
C007	4.0	1.0	5.0	2.972	.040	1.102	1.214
C008	4.0	1.0	5.0	2.898	.041	1.131	1.279
**D -** School policies and leadership toward the implementation of the STEAM project							
D001	4.0	1.0	5.0	2.512	.038	1.051	1.105
D002	4.0	1.0	5.0	2.633	.040	1.096	1.202
D003	4.0	1.0	5.0	2.618	.040	1.092	1.193
D004	4.0	1.0	5.0	2.422	.040	1.083	1.174
D005	4.0	1.0	5.0	2.633	.040	1.101	1.213
D006	4.0	1.0	5.0	2.507	.039	1.074	1.153

Project-based learning allows children to engage with real-life issues through various individualized learning experiences, including observation, inquiry, and hypothesis testing, as a team or as an individual [8]. The project-based learning approach in developing STEAM programs for preschool children has shown its superiority in capacity development for preschool children in the 21st century [9]. In addition, under the prism of place-based education, integrating local cultural values, materials, or practices also enhances the engagement of children's learning processes [10,11]. Especially, those integrated activities even help to strengthen children's storytelling skills, cultural awareness, and cultural responsiveness [12]. Table 4 presents Vietnamese preschool teachers' evaluation of the ease and their willingness to integrate tangible (E001 to E005) and intangible (E006 to E011) cultural aspects into STEAM activities (F001, F002) and projects (F003, F004).

**Table 4:** Descriptive statistic of Local Culture integration in STEAM projects and Teacher's readiness to implement STEAM activities/ projects for preschoolers (*N* = 754).

Variables	Range	Min	Max	Mean	Std. Error	Std. Deviation	Variance
**E –** Local culture factors integrated with STEAM projects							
E001	4.0	1.0	5.0	2.732	.044	1.215	1.477
E002	4.0	1.0	5.0	2.387	.042	1.145	1.311
E003	4.0	1.0	5.0	2.820	.043	1.166	1.359
E004	4.0	1.0	5.0	2.891	.043	1.182	1.396
E005	4.0	1.0	5.0	2.934	.043	1.182	1.398
E006	4.0	1.0	5.0	2.858	.042	1.158	1.341
E007	4.0	1.0	5.0	2.972	.042	1.165	1.358
E008	4.0	1.0	5.0	2.809	.042	1.153	1.329
E009	4.0	1.0	5.0	2.496	.043	1.174	1.379
E010	4.0	1.0	5.0	2.580	.042	1.159	1.344
E011	4.0	1.0	5.0	2.940	.042	1.162	1.350
**F –** Teachers' willingness to implement STEAM activities/ projects for preschoolers							
F001	3.0	1.0	4.0	3.180	.023	.633	.400
F002	3.0	1.0	4.0	3.143	.024	.644	.415
F003	3.0	1.0	4.0	3.160	.024	.654	.427
F004	3.0	1.0	4.0	3.115	.025	.675	.455

The survey results were analyzed using correlation analyses. Table 5 shows the correlation between two constructs: (i) preschool teachers' understanding of STEAM education (No. from A001 to A009) and (ii) their competencies in implementing STEAM activities and projects (No. from B001 to B017).

**Table 5:** Correlations table for survey questions.

Variables	A001	A002	A003	A004	A005	A006	A007	A008	A009
B001	.594**	.561**	.542**	.555**	.517**	.482**	.575**	.568**	.577**
B002	.561**	.563**	.529**	.533**	.518**	.485**	.542**	.547**	.535**
B003	.582**	.555**	.525**	.543**	.518**	.489**	.585**	.589**	.580**
B004	.542**	.574**	.558**	.549**	.535**	.515**	.554**	.551**	.548**
B005	.549**	.556**	.527**	.527**	.507**	.474**	.565**	.553**	.557**
B006	.525**	.556**	.501**	.506**	.534**	.500**	.523**	.506**	.514**
B007	.533**	.554**	.523**	.494**	.546**	.503**	.527**	.516**	.532**
B008	.534**	.561**	.511**	.496**	.528**	.514**	.509**	.506**	.497**
B009	.529**	.575**	.532**	.503**	.537**	.522**	.504**	.506**	.517**
B010	.544**	.571**	.536**	.502**	.544**	.531**	.514**	.506**	.507**
B011	.529**	.570**	.536**	.510**	.532**	.520**	.524**	.517**	.508**
B012	.524**	.537**	.510**	.495**	.519**	.479**	.516**	.507**	.512**
B013	.527**	.556**	.532**	.498**	.521**	.483**	.515**	.509**	.521**
B014	.523**	.560**	.521**	.490**	.526**	.513**	.514**	.511**	.519**
B015	.508**	.552**	.516**	.494**	.528**	.524**	.500**	.506**	.496**
B016	.550**	.580**	.545**	.528**	.534**	.529**	.540**	.527**	.524**
B017	.536**	.568**	.539**	.518**	.529**	.510**	.534**	.521**	.528**

Note: * p < .05 (2-tailed); ** p < .01 (2-tailed)

## 2 Experimental Design, Materials, and Methods

To design the research approach, we first referred to the systematic review of Wan et al. [13], which outlined the demand to measure preschool teachers' perspectives and capacity to implement STEM lessons, as well as the involvement of related stakeholders and policies. The lower the self-efficacy of a teacher, the less chance they will decide to design and implement STEAM activities or projects. Therefore, this dataset focuses on revealing Vietnamese preschool teachers' perceptions, competencies, and willingness to implement STEAM activities and projects. Besides, we also present the readiness of Vietnamese preschools towards STEM education regarding infrastructure and principals' support. In addition, we also discuss the concerns of integrating local cultural content into STEAM activities.

This data set mainly clarified the dependence of the willingness to implement STEAM education activities for preschool children with related aspects: Self-Knowledge (KNO); own proficiency level (COM); operating conditions (FAC); the level of support for implementing STEAM education projects and the ability to integrate local cultural factors (CUL). For instance, an overall regression model that illustrates the relationship between those above factors and preschool teachers' willingness to implement STEAM activities for preschoolers can be formed as follows:

$$\text{WIL}∼\beta_{0}+\beta_{1}*\text{KNO}+\beta_{2}*\text{COM}+\beta_{3}*\text{FAC}+\beta_{4}*\text{LEA}+\beta_{5}*\text{CUL}+u$$

We examined factors that support and challenge all teachers in formulating questions regarding the willingness to organize STEAM activities/ projects for preschoolers. The first aspect (KNO) includes the Teacher's understanding of the concept of STEAM, educational content in each S-T-E-A-M field, how to integrate STEAM topics and activities, Teaching methods in STEAM education, and support resources to be explored. The second aspect (COM) includes preparation (selecting topics, defining objectives, content, and planning), environment design (arranging research objects, designing equipment, learning tools, etc.), mobilize materials and toys from different sources, build a psychological environment), organize activities (asking questions; observing children capture child's interest; stimulating children to investigate; explore, attract the participation of educational forces; use of technology), assessment (process assessment; product evaluation; self-assessment). The third aspect (FAC) includes time (prep time; time for carrying out STEAM activities); quantity and quality of toys; reference materials; the number of children in a class; and time for organizing activities. The fourth factor (LEA) includes time to study, organize activities/projects, support from forces inside and outside of school, motivation for teachers to implement STEAM projects, and funding for STEAM projects. The last aspect (CUL) concludes with eleven local cultural characteristics such as historical-cultural relics, scenic spots, folklore (old stories, folk songs, proverbs...), crafts, culinary, etc. Besides the traditional approaches such as ordinal least square regression, ANOVA (to compare the outputs of various participant groups), and SEM (to explore the inter-relationship between factors), this dataset is also suitable to construct new conceptual frameworks of ECE teacher development, using BMF (Bayesian Mindsponge Framework) [14].

## Ethics Statements

Participation in this dataset is fully anonymized and voluntary. Informed consent was obtained from all participants before they participated in this study. The work was conducted with the approval of the Hanoi National University of Education's Institutional Review Board, Decision No. 134/GCN-ĐHSPHN on 22 Apr 2022.

## CRediT authorship contribution statement

**Thi-Lam Bui:** Conceptualization, Supervision. **Thi-Tham Tran:** Investigation, Software. **Thanh-Huong Nguyen:** Investigation, Methodology. **Luyen Nguyen-Thi:** Methodology, Investigation. **Viet-Nhi Tran:** Investigation, Software, Writing – original draft. **Ut Phuong Dang:** Investigation, Methodology. **Manh-Tuan Nguyen:** Investigation, Writing – original draft, Data curation. **Anh-Duc Hoang:** Conceptualization, Writing – review & editing.

## Declaration of Competing Interest

The authors declare that they have no known competing financial interests or personal relationships that could have appeared to influence the work reported in this paper.

## Data Availability

Replication data for “Develop STEAM education projects for Vietnamese preschool children based on local cultural aspect” (Original data) (Harvard Dataverse)
